# Determination of 1-Deoxynojirimycin (1-DNJ) in Leaves of Italian or Italy-Adapted Cultivars of Mulberry (*Morus* sp.pl.) by HPLC-MS

**DOI:** 10.3390/plants10081553

**Published:** 2021-07-28

**Authors:** Lucia Marchetti, Alessio Saviane, Antonella dalla Montà, Graziella Paglia, Federica Pellati, Stefania Benvenuti, Davide Bertelli, Silvia Cappellozza

**Affiliations:** 1 Department of Life Sciences, University of Modena and Reggio Emilia, Via G. Campi 103, 41125 Modena, Italy; lucia.marchetti@unimore.it (L.M.); federica.pellati@unimore.it (F.P.); stefania.benvenuti@unimore.it (S.B.); 2 Doctorate School in Clinical and Experimental Medicine (CEM), University of Modena and Reggio Emilia, 41125 Modena, Italy; 3 Consiglio per la Ricerca in Agricoltura e L’Analisi Dell’Economia Agraria (CREA)-Centro per la Ricerca Agricoltura e Ambiente, Laboratorio di Gelsibachicoltura, Via Eulero, 6a, 35143 Padova, Italy; alessio.saviane@crea.gov.it (A.S.); antonella.dallamonta@crea.gov.it (A.d.M.); graziella.paglia@crea.gov.it (G.P.); silvia.cappellozza@crea.gov.it (S.C.)

**Keywords:** *Morus* sp. pl., cultivar, mulberry, 1-DNJ, HPLC-ESI-MS, HILIC

## Abstract

Recently, 1-DNJ has been widely studied by scientists for its capacity to inhibit α-glucosidase and reduce postprandial blood glucose and fat accumulation. To the best of our knowledge, this is the first analytical determination of 1-DNJ in *Morus* sp.pl. leaves carried out on Italian crops, and it could be used as a reference to assess the quality of the plant material in comparison to Far Eastern Asia cultivations. The effects of two thermal treatments were compared to test the incidence of the drying process on the 1-DNJ extractability. In addition, two harvesting seasons in the same year (2017) and two subsequent harvesting years (2017–2018) were considered. The amount of 1-DNJ herein found was comparable to that reported in the scientific literature for Asian cultivations. The increase in 1-DNJ along the summer and the higher level of this compound in the apical leaves also complies with previous findings. However, a strong implication for the climatic conditions in the different years and a significant interaction between climate and genotypes suggest exploring very carefully the agronomic practices and selecting cultivars according to different environmental conditions with a view to standardize the 1-DNJ amount in leaves.

## 1. Introduction

Some peculiar characteristics of the third millennium in the Old Continent can be summarized in a few words: increased life expectancy, ageing population, and wide media coverage of health-related topics. Consequently, Europe is a promising market for natural ingredients and companies are continuously launching new natural health products; on the other hand, European consumers are very sensitive to any advertising regarding complementary and alternative medicine. These trends are not expected to change soon so that demand for pharmaceuticals, supplements, and alternative medicine products are forecast to increase in the European market as well as the demand for nutraceuticals. These substances are capable of giving physiological benefits or providing protection against chronic diseases [1]. Among natural products extracted from plants and other sources, iminosugars are analogs of sugars in which an atom of nitrogen replaces the ring oxygen atom. This substitution results in the inhibition of glycosidases and glycosyltransferases, therefore preventing normal carbohydrate metabolism [2]. The compound 1-Deoxynojirimycin (1-DNJ) is one of the most popular iminosugars and was first extracted from mulberry (*Morus* sp.pl.) roots in 1976 [3]. This active ingredient, along with others, interferes with sugar metabolism, and was found at surprisingly high concentrations in the mulberry leaf latex. Indeed, it is involved in the mechanism of defense against insects, as the mulberry leaves are highly toxic for most species of Lepidopterans others than *Bombyx mori* [4]. Even though active ingredients contained in the mulberry leaf are harmful to herbivorous insects, man learnt to use their therapeutic effect from ancient times [5,6]. Furthermore, from the end of the last century 1-DNJ has been widely studied by scientists for its capacity to inhibit α-glucosidase and tumor cell metastasis, reducing postprandial blood glucose and fat accumulation, contrasting aging-related behaviors and HIV progression [7]. Due to its powerful and wide range of possible applications in therapeutics, various efforts have also been carried out to identify the highest-yielding mulberry varieties [8,9,10,11,12], the richest branch portions in 1-DNJ leaf content [8,9,11,13], the optimal harvesting season [14], the influence of the cultivation area and its interaction with the mulberry varieties [14], in addition to the best leaf processing and extraction method [7,9,15]. Furthermore, as mulberry leaves are the only feed of the silkworm *B. Mori*, which is efficient at concentrating the active ingredient in its body tissues, researchers have also been investigating this insect as a precious bio-accumulator [7,16,17]. However, most current research on 1-DNJ has been carried out in Far Eastern Asia (China, India, Korea, Thailand, and Japan), while few data are available on the content of 1-DNJ in the mulberry cultivars used in Europe. Therefore, in the current paper, we tried to fill this gap of knowledge with regard to the European genetic material, by also taking already-tested Japanese mulberry cultivars, which were grown under European conditions, as a reference. Furthermore, since the correlation among the cultivation area, the genetic material, and the environmental conditions (season of harvesting) has already been proven [14], we repeated the test on different cultivars in two subsequent years. The aim was to ascertain whether the difference in the 1-DNJ level, although variable in absolute value along the years, would respect the same ranking, being affected by the plant genetic constitution more than environmental conditions. Another aspect we took into consideration was the treatment of the collected material (leaf) before extraction, which was freezing of fresh samples or drying. In fact, regarding this aspect information is not very exhaustive because it is generally accepted that 1-DNJ is quite resistant to high temperatures [9,18]; but this topic has not been definitely tested.

Different methods for the determination of 1-DNJ are available in the literature, and other quantitative techniques are constantly being developed. Regardless, some issues have to be considered from an analytical point of view. First, due to the high hydrophilicity and small molecular weight of this compound, the interaction with the stationary phase of conventional reversed phase columns is so weak that 1-DNJ is not retained in the column. Moreover, since the molecule lacks a chromophore, direct UV detection is not suitable like for many other aminoglycosides. Some authors reported pre-column derivatization methods, which, however, involve sample manipulation and ultimately may result in the introduction of impurities or degradation products [19]. On the other hand, derivatization is unnecessary for an HPLC-evaporative light scattering detector (HPLC–ELSD) and HPLC-tandem mass spectrometry (HPLC–MS/MS). Kimura et al. successfully used an evaporative light scattering detector (ELSD), which is a universal detector, for relatively non-volatile analytes [20]. The main drawbacks are the poor sensitivity and the long time required to complete the analysis. Hydrophilic interaction chromatography (HILIC), coupled with mass spectrometry, has proven to be a promising technique for 1-DNJ quantitation. In our opinion, this is the most convenient method since it showed the lowest LOD and LOQ and the shortest retention time.

## 2. Results and Discussion

**Figure 1:**
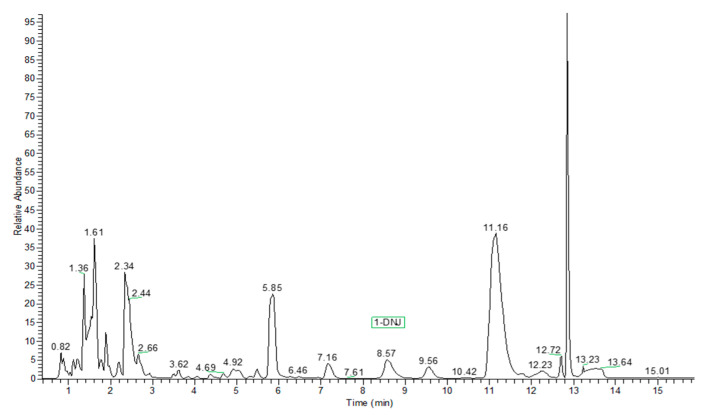
Typical base peak chromatogram of mulberry leaf hydroalcoholic extract.

**Figure 2:**
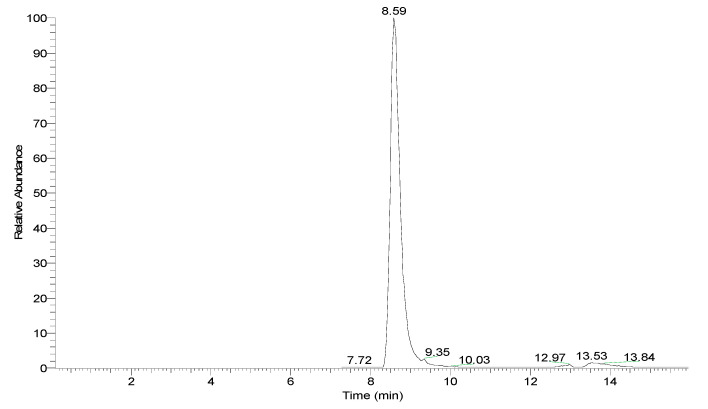
Base peak chromatogram of 1-DNJ standard solution (6.67 ppm).

The herein used analytical method employs hydrophilic interaction chromatography (HILIC). The hybrid quadrupole-orbitrap mass parameters were optimized under positive ion electrospray ionization. The compound 1-Deoxynojirimicin was detected by MS/MS with parallel reaction monitoring (PRM) for transition of the parent ion to the product ions. In the reported experimental conditions, retention time (t_R_) of 1-DNJ was 8.57 min, with minimal shifts between samples (Figure 1). The chromatogram of 1-DNJ standard solution (6.67 ppm) is shown in Figure 2.

**Figure 3:**
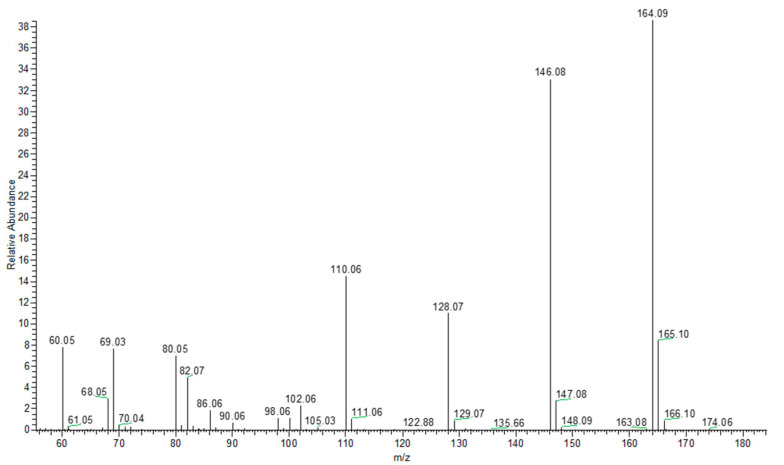
ESI mass spectrum of 1-DNJ.

Figure 3 shows 1-DNJ mass spectrum; an intense molecular ion [M+H]^+^ *m*/*z* 164.0919 and other intense fragment ions generated by the consecutive loss of water molecules are present: *m*/*z* 60.0453 (8), 69.0343 (8), 80.0502 (8), 110.0604 (14) [M+H−3H_2_O]^+^, 128.0708 (11) [M+H−2H_2_O]^+^, 146.0812 (33) [M+H−H_2_O]^+^, 164.0919 (100). The integral of the product ion peak (*m*/*z* 146.0811) was used for the quantification.

**Figure 4:**
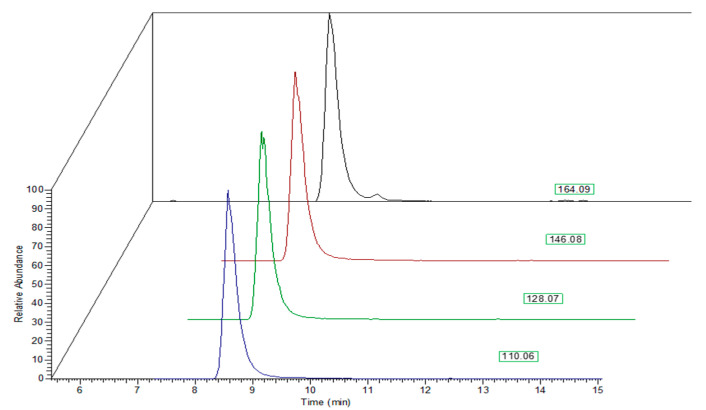
Chromatograms of extracted ion peaks used for quantification of 1-DNJ and as qualifier ions.

The residual precursor ion and other fragment peaks were used as qualifier ions (Figure 4).

Nakagawa et al. previously applied HILIC–MS/MS for the simultaneous determination of DNJ and its derivatives. In this case, the selective detection of DNJ was achieved by monitoring the transition *m*/*z* 164 to 69 [M+H−95]^+^ [21], thus indicating that MRM was adaptable for the HILIC–MS/MS analysis of iminosugars in general. During recent decades, HILIC has proven to be an efficient strategy for the analysis of highly polar compounds, including sugars and peptides. Moreover, the presence of amino groups is prone to the elongation of retention time [22]. Under the herein optimized conditions, DNJ was clearly detected at a reasonable retention time.

Data in Table 1, Table 2 and Table 3 are expressed as the overall mean of two sample replicates, each analyzed twice, ±SD (standard deviation). Data were obtained from the analysis of dried leaves and are expressed as 1-DNJ mg/g dry weight (DW). The content of 1-DNJ was determined in both fresh frozen and dried leaves in order to evaluate the influence of the drying process (50 °C) on the active compound extractability, and to assess the potential loss due to thermal degradation. Indeed, we tried to assess if the preservation of the mulberry leaf (freezing or drying) is influent on the extractable DNJ content. With this aim, a representative portion of frozen leaves was ground in an automatic mill and extracted with ethanol 50%. The same procedure was repeated on dried leaves.

Therefore, data reported in Table 1 are shown in comparison with the 1-DNJ content obtained from fresh leaves and then corrected by the value of humidity related to the specific cultivar.

Table 1 shows the 1-DNJ contents of 31 cultivars from different *Morus* species, harvested at the end of May 2017. The object of the study was not to identify the compositional characteristics of the different varieties in terms of secondary metabolites and iminosugars. The purpose of the current research was to screen among the most spread varieties largely represented in Italy (country taken as an example of the Mediterranean area) and used for sericulture or fruit production, to enlarge the chance of exploiting the already existing mulberry fields for the pharmaceutical industry. The relative percentages of humidity ranged from 52.9 (Egyptienne, *M. alba* L.) to 74.4% (Nervosa, *M. alba* L.). Two-way ANOVA was carried out by taking into consideration two main factors (thermal treatment and cultivars) and their interaction. The statistical analysis confirmed a significant difference in the 1-DNJ content among the different cultivars at *p* < 0.05, and a better 1-DNJ yield in the fresh-frozen samples in comparison to the dried ones at *p* < 0.05. The average 1-DNJ loss in dried samples was 12.8%. Only in two cases (Chirtut and Illinois everbearing) an increase in the iminosugar content was observed. This slight variation in the DNJ content might be attributable to random sampling or the analysis error; however, it is worthy of noting that it occurred in the only two varieties belonging to species other than *M. alba*. Therefore, it might be due to a different leaf morphology (hairs, waxes, other leaf structures, thickness…) or physiology (enzymatic reactions). The interaction between the two factors (cultivar and thermal treatment) was also significant. The post hoc Tukey’s test following ANOVA analysis of the different cultivars demonstrated that a great variation was present in the genetic material. Table 1 reports cultivars and their species; the genus *Morus* shows a very complex and uncertain taxonomy because of its broad geographical distribution, long domestication history, morphological plasticity, and documented inter-species hybridization [23]; according to Nepal and Ferguson [24] it comprises 10–13 species, but Zeng et al. recently identified eight species only: *M. alba*, *M. nigra*, *M. notabilis*, *M. serrata*, *M. celtidifolia*, *M. insignis*, *M. rubra*, and *M. mesozygia*, based on the technique of internal transcribed spacer-based phylogeny [25]. Therefore, according to this classification, cultivars reported in Table 1 all belong to *M. alba*, except for Hicks fancy, which is ascribable to *M. rubra*, for the sample of *M. nigra* wild accession, which does not belong to any selected cultivar, and for the *M. nigra* cultivar Chirtut. It is quite clear that the variation in the 1-DNJ content is as broad among cultivars belonging to the same species as it is among different species.

Since the drying procedure was undertaken at 50 °C, far below the degradation limit temperature, the hypothetical heat-due deterioration of the compound determined in dried leaves seems unlikely. The explanation for the phenomenon is likely simple: in fact, we should take into account the worsening of the compound extractability according to the reduction of the water content, a condition that occurs with the loss of moisture during desiccation [26]. However, it should also be considered that low temperatures (below 50 °C) allow time for enzymatic conversions and respiratory losses, whereas high temperatures (above 80 °C) can cause thermochemical degradation [27]. Therefore, it is worth exploring the difference between short thermal treatments at higher temperatures and longer thermal treatments at lower temperatures to analyze how the duration of the desiccation process affects the 1-DNJ stability in the leaf, especially in the first drying phase, when the water content is still considerable. Furthermore, an interaction between the two factors (thermal treatment and CVs) was demonstrated (*p* < 0.05). This interaction was found to be particularly significant for Restelli (*M. alba* L.), Gorgeous (*M. alba* L.), Romana lhou (*M. alba* L.) Wildtype (*M. nigra* L.), and Korin (*M. alba* L.). In these cases, the 1-DNJ decrease in dry leaves was remarkable. The morphology of the leaf surface, the wax content, the thickness of the leaf blade and leaf hairs can otherwise affect the drying process, determining different efficiencies in the preservation of the compound.

Hu et al. previously investigated the content of 1-DNJ in mature leaves from 132 *Morus* cultivars belonging to nine Chinese species; among them, *M. alba* was the most represented. The mean content found by the authors ranged from 0.13 to 1.47 mg/g DW [28]; these data are slightly lower with respect to the results herein discovered, in which the concentration of 1-DNJ ranged from 0.44 mg/g (Aobanezumi) to 7.07 mg/g (Korin). In addition, comparing the values for Kayriounezumigaeshi and Aobanezumi, recovered under our conditions with the ones shown by Kimura et al. [9], we can observe a higher 1-DNJ level for Kayriounezumigaeshi, but a lower level for Aobanezumi, in May harvests of both years. This fact highlights that the cultivar adaptation to different climatic situations can greatly affect the 1-DNJ content in leaf samples.

Table 2 reports the 1-DNJ content found in 14 *M. alba* cultivars, relative to the two harvests of 2018. The mean humidity percentages recovered were 66.2 ± 5.8 and 64.9 ± 6.4 in May 2018 and July 2018, respectively. The slight diminution in water in the leaf is a marker of the advancement of leaf maturation; young leaves are richer in water, but their content in nutrients is still low. Pendula was the richest in 1-DNJ both at the end-May and the end-July 2018 harvests. As is well known and extensively outlined in the literature, weather conditions and harvest time play a key role in the abundance of active substances in *Morus* sp. pl. [28,29]. Typically, iminosugar levels start increasing in April, and then the concentration gradually decreases until September, with the maximum around June and July [29]. This study was a first test aimed at investigating the seasonal fluctuation of the DNJ content in leaves even in the Mediterranean area and on local (Italian) varieties, which were compared to Japanese varieties, already considered by other authors.

This trend was also observed in data reported in Table 2. In fact, the mean content of 1-DNJ was 0.68 ± 0.23 mg/g and 2.39 ± 1.69 mg/g in the end-May 2018 and the end-July 2018 harvest, respectively. Hence, the mean 1-DNJ content increased more than threefold, starting from May to July. The two-way ANOVA performed considering simultaneously all the CVs and the two seasons assessed the significance of differences between the two seasons and among CVs (*p* < 0.05). The post hoc test showed various homogeneous subsets of CVs were identifiable (lowercase letters in columns). It must be observed that the correlation between seasons and CVs was also significant (*p* < 0.05). Different factors can contribute to this mulberry plants response to the different seasons: first of all, the amount of the average monthly radiation, which, under the conditions of the North-Eastern part of Italy, increases from May to July; the total radiation received by the canopy of mulberry plants might have a direct effect on photosynthesis and, therefore, on the iminosugars synthesis. The same trend is recorded for the average daily temperature, which is also important for plant metabolism. Furthermore, DNJ synthesis by plants might be the defense reaction to the attack of insect populations (increasing their density with subsequent generations in summer), as this iminosugar shows remarkable biological activity against herbivorous predators, by inhibiting intestinal α-glucosidase [30].

Our findings are not conclusive: in fact, the difference in the DNJ-1 content in the leaves of the Italian Cultivars (Cattaneo female and male, Florio, Giazzola, Limoncina, Morettiana, Nervosa, Pendula, Pyramidalis, Spain Black Fruit) should have been tested in the two different seasons both in 2017 and 2018, while data are available for 2018 only. However, our one-year data collection agrees with those of other authors [9,31,32].

**Figure 5:**
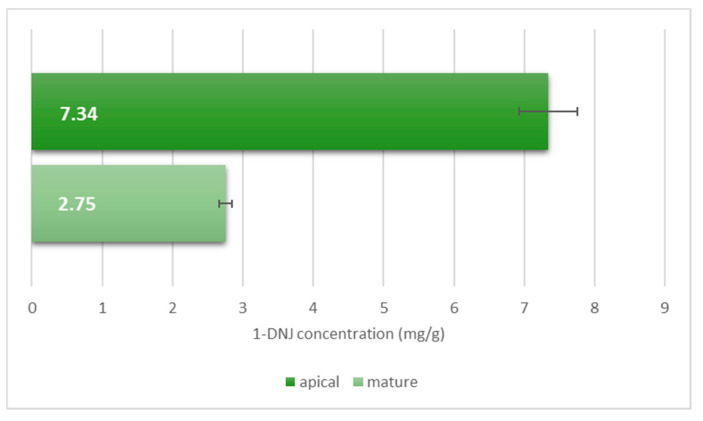
1-DNJ content by leaf-maturity type in cultivar Morettiana.

In addition, in the end-July 2018 harvest, Morettiana was selected and analyzed both in the form of apical leaves and branch mature leaves to study the distribution of the constituent in the different parts of the plant. The cultivar Morettiana was specifically chosen because this is one of the most representative and widespread Italian cultivars. The 1-DNJ content of the younger leaves (data not shown in Table 2) was equal to 7.34 ± 0.42 mg/g of dry matter (Figure 5).

These data are consistent with what was previously observed by Hu et al. [28], establishing that apical younger leaves usually contain higher 1-DNJ than mature ones. This fact is strengthened by the major enzymatic activity discovered in young leaves that is responsible for the imination of glucose, the initial step in 1-DNJ biosynthesis [12,33].

In Table 3, data from 2017 and 2018 spring harvests are compared. The two-way ANOVA performed, considering CVs and harvesting year simultaneously, showed a significant effect for the two main factors CVs (*p* < 0.05) and year (*p* < 0.05), and a significant interaction among factors, demonstrating the importance of annual variation in 1-DNJ content, which could be influenced by annual climatic fluctuations. In particular, the amount of 1-DNJ, which was recorded in the mulberry leaf samples at the end of May 2017 was almost double in comparison to the amount in those collected at the end of May 2018. The climatic conditions in the two years were very different: in the Padua province, North-Eastern Italy, where the experiment was carried out, May 2018 was rainier (81.8 mm) than May 2017 (79 mm) [34].

Furthermore, as it is possible to observe in Table 4, the average daily accumulated energy (calculated at the ground level on the horizontal surface) was 5.16 kWh/m^2^/day (18.57 MJ/m^2^/day) in May 2018 while it was 5.70 kW/m^2^/day (20.52 MJ/m^2^/day) in May 2017 [34]. It can be assumed that higher solar radiation (recorded in End-May 2017) produced increased photosynthetic activity and 1-DNJ biosynthesis. In addition, due to the humid conditions and high average temperature (May 2018 average monthly temperatures was 19.4, while May 2017 average monthly temperature was 18.6), fungal attacks spread on the mulberry plants, affecting the leaf surface, and by extension the photosynthetic capacity. The methodology used to produce these meteorology and solar radiation data are available online [35].

In conclusion, this work aims at the investigation of the 1-DNJ amount in leaves of different CVs of mulberry grown in Italy, and at understanding its seasonal fluctuations, with a view to determining a rational harvesting time. To the best of our knowledge, this is the first analytical determination of 1-DNJ carried out on Italian mulberry crops. This study could be used as a reference to assess the quality of Italian plant material in comparison to cultivations carried out in Far Eastern Asia. Based on our data, we can conclude that the best harvesting time is in the middle of summer rather than in springtime, and that apical young leaves are much richer in 1-DNJ than older ones at the base of the shoot. However, with regard to the cultivar selection for the 1-DNJ production, agronomical tests in subsequent years and under different cultivation conditions are recommended. Indeed, the interactions with the weather conditions are determinant for the 1-DNJ leaf content, which, in turn, depends on the physiological status of the tree and its photosynthetic capacity. Furthermore, the drying procedure should be optimized for industrial use before upscaling the crop for pharmaceutical exploitation to avoid the loss of important compounds as well as thermal inactivation during leaf desiccation. The results of the recovery and precision studies are shown in Table 5.

## 3. Materials and Methods

### 3.1. Chemicals and Solvents

All solvents were analytical grade and purchased from Sigma-Aldrich (Milan, Italy). The reference standard of 1-DNJ (purity ≥ 95.0%) was purchased from Sigma-Aldrich (Milan, Italy). Water was purified by using a Milli-Q Plus185 system from Millipore (Milford, MA, USA).

### 3.2. Plant Material and Extraction Procedure

*Morus* sp.pl. (L.) leaves were harvested at different times, (end-May 2017, end-May 2018, and end-July 2018), and were provided by CREA—Research Centre for Agriculture and Environment, laboratory of sericulture (Padova, Italy). This experimental mulberry field preserves a germplasm bank of around 60 *Morus* cultivars. The first screening was carried out by considering the more representative cultivars of the collection (the varieties of different geographic origin and the same age and pruning type, which were therefore comparable in this study). Within this group, for the subsequent analyses, a more limited number of varieties was explored; these varieties are the most used for sericulture or fruit production in the country, and therefore easily exploitable for pharmaceutical uses too.

To ensure the uniformity of the sampling, leaves were collected randomly from at least five different trees per cultivar. A representative sample of about 250 g was labelled in plastic bags and stored according to the chosen treatment method. The content of 1-DNJ was determined in both frozen and dried leaves in order to evaluate the influence of the drying process on the active compound extractability and to assess the potential loss due to thermal degradation. *Morus* leaves were weighed as fresh samples; afterwards, a mild drying process was carried out in an oven at 50 °C until reaching constant weight; then the percentage of humidity was calculated per each sample. Samples were stored at −18 °C and protected from light by storage inside amber glass sealed containers. Then, to prepare the fresh samples, with the aim to prevent any oxidation or degradation process, a representative portion of frozen leaves was ground in an automatic mill with dry ice. Immediately after, two grams of powdered frozen leaves were extracted at room temperature with 20 mL of a water:ethanol (50:50) solution under magnetic stirring. The whole procedure required three consecutive extraction steps of 2 h each. The first extract was centrifuged (RCF 7200 g) for 5 min, the supernatant was collected, paper filtered, and the further two extractions were performed on the residue. The three extracts were combined, and the final volume was adjusted to 100 mL with the same hydroalcoholic solution; the extract was then stored at 4 °C in flasks covered with silver paper until analysis. The extraction procedure was repeated twice for each sample, and it was performed in the same way for dried samples too. Before injection in the UHPLC system, the extracts were filtered by using a 0.22 μm cellulose acetate filter (GVS, Bologna, Italy) into an HPLC vial and properly diluted with acetonitrile.

### 3.3. HPLC-ESI-MS Analysis

The chromatographic method previously developed by Vichasilp et al. [36], was herein properly modified and optimized. A Thermo Scientific Dionex Ultimate 3000 UHPLC system equipped with an autosampler, a quaternary pump, and a thermostated column compartment controlled by Chromeleon 7.2 Software (Thermo Scientific, Waltham, MA, USA) was used. The UHPLC system was coupled with a high-resolution Q Exactive mass spectrometer (Thermo Scientific, Bremen, Germany). The mass spectrometer was calibrated before the analyses. Nitrogen (N_2_) (purity > 99.99%), obtained from a Zefiro zero 60 LC-MS nitrogen generator (CINEL, Vigonza, Italy), was employed both as the source gas and the collision gas. The capillary temperature was set at 270 °C and the following N_2_ flows (arbitrary units) were used: sheath gas 40, auxiliary gas 30, and sweep gas 3. The MS/MS parameters were optimized with standard 1-DNJ under positive ion electrospray ionization. A Hydrophilic Interaction Liquid Chromatography column (Cortecs UPLC HILIC, 1.6 μm, 2.1 × 100 mm) was used (Waters, Milford, MA, USA). This kind of column provides strong retention of very polar molecules that are typically unretained under conventional reversed phase conditions. 1-DNJ was eluted with a binary gradient consisting of ammonium formate 20 mM in water (solvent A) and acetonitrile (solvent B). The gradient profile was as follows: 0–10 min, 10% A; 11–16 min, 50% A; 16–27 min, 10% A. The flow rate was adjusted to 0.3 mL/min, and the column temperature was maintained at 30 °C. 1-Deoxynojirimycin was detected by MS/MS with Parallel Reaction Monitoring (PRM) for transition of the parent ion to the product ions. The concentration of 1-DNJ in samples was obtained through the calibration curve built with the pure standard compound. The selected range was 0.31 to 40.9 mM, corresponding to 0.050–6.67 ppm.

### 3.4. Method Validation

The method showed good linearity (*r*^2^ > 0.9995) within the selected range of concentrations. The method was validated in terms of precision and accuracy. Precision was evaluated with the relative standard deviation determined from repeated extract injections and triplicate extractions. Method accuracy was evaluated by recoveries, which were carried out by spiking samples with three different concentrations of standard solutions. Spiking recovery (%) was calculated as: [(C_2_ − C_0_)/C_1_] × 100, where C_2_ is the analyte concentration in the final solution after spiking with a known concentration of standard, C_0_ is the original analyte concentration in the initial solution, and C_1_ is the added known concentration of standard. Intra-day precision was determined by analyzing three replicates of spiked samples, and inter-day precision was determined by running the three replicates of spiked samples on three different days.

### 3.5. Statistical Analysis

Statistical analysis was carried out by using Statistica 6.0 (StatSoft Italia, Vigonza, Italy) and SPSS 13.0 (SPSS Inc., IBM, Segrate, Italy), the significance was established at *p* < 0.05. One-way ANOVA or factorial ANOVA was carried out according to the experimental situation. In addition, post hoc analysis based on Tuckey’s HSD test was performed to evaluate differences among means.

## Figures and Tables

**Table 1:** 1-DNJ content of 31 cultivars from five different *Morus* species harvested at the end of May 2017. Data are expressed as mg of 1-DNJ/g of dry leaf ± SD. Dry samples data are shown in comparison with fresh samples corrected by the relative humidity.

Cultivar	End-May 2017		
	Humidity (%)	Fresh Samples (mg/g of Dry Matter) ^a^	Dry Samples (mg/g of Dry Matter) ^a^
Aobanezumi (*M. alba* L.)	69.4	0.55 ± 0.02 m,n	0.44 ± 0.06 z
Cattaneo female (*M. alba* L.)	66.3	0.66 ± 0.02 m,n	0.59 ± 0.03 z,v
Cattaneo male (*M. alba* L.)	70.5	1.87 ± 0.02 g,h,i	1.50 ± 0.03 o,p,q
Egyptienne (*M. alba* L.)	52.9	1.78 ± 0.04 g,h,i	1.66 ± 0.08 o,n
Florio (*M. alba* L.)	70.7	0.74 ± 0.02 n,m	0.55 ± 0.02 v,z
Giazzola (*M. alba* L.)	67.7	1.33 ± 0.05 i,l	1.20 ± 0.05 s,t
Kayriounezumigaeshi (*M. alba* L.)	68.1	1.99 ± 0.04 g,h	1.86 ± 0.08 l,m
Limoncina (*M. alba* L.)	64.2	1.01 ± 0.02 l,m	0.93 ± 0.05 u
Morettiana (*M. alba* L.)	72.5	1.36 ± 0.02 i,l	1.26 ± 0.04 r,s
Nervosa (*M. alba* L.)	74.4	1.70 ± 0.04 h,i	1.62 ± 0.06 n,o,p
Pendula (*M. alba* L.)	72.6	1.49 ± 0.05 h,i,l	1.43 ± 0.04 q,r
Pyramidalis (*M. alba* L.)	70.2	2.19 ± 0.06 g	2.07 ± 0.08 i
Spain Black fruit (*M. alba* L.)	70.3	1.81 ± 0.02 g,h,i	1.76 ± 0.07 m,n
Ukraina (*M. alba* L.)	70.5	2.99 ± 0.03 f	2.94 ± 0.06 f
Filippine (*M. alba* L.)	65.9	2.26 ± 0.11 n	2.22 ± 0.11 h
Kokosou 21 (*M. alba* L.)	70.4	3.17 ± 0.11 f	2.50 ± 0.04 g
Restelli (*M. alba* L.)	72.3	4.56 ± 0.11 d	3.45 ± 0.05 e
Gorgeous (*M. alba* L.)	70.6	5.65 ± 0.11 c	4.24 ± 0.01 d
Queensland black (*M. alba* L.)	64.4	3.56 ± 0.11 f,e	2.96 ± 0.02 f
Romana lhou (*M. alba* L.)	70.7	8.55 ± 0.11 b	6.86 ± 0.22 b
Wildtype (*M. nigra* L.)	71.9	5.99 ± 0.11 c	4.46 ± 0.04 c
Chirtut (*M. nigra* L.)	55.2	0.56 ± 0.11 m,n	0.66 ± 0.03 v
Date-Akagi (*M. alba* L.)	68.7	1.61 ± 0.11 h,i	1.45 ± 0.21 p,q
Akagi (*M. alba* L.)	61.2	1.57 ± 0.11 h,i	1.22 ± 0.03 s
Illinois everbearing (*M. rubra* L.)	59.8	1.74 ± 0.11 g,h,i	1.96 ± 0.02 i,l
Muki (*M. alba* L.)	69.5	5.66 ± 0.11 c	4.49 ± 0.03 c
Korin (*M. alba* L.)	72.2	9.55 ± 0.11 a	7.07 ± 0.14 a
Miura (*M. alba* L.)	70.7	4.98 ± 0.1 d	4.32 ± 0.03 c.d
Platanoide (*M. alba.*)	69.0	3.14 ± 0.11 f	2.57 ± 0.02 g
Sinuense (*M. alba* L.)	67.5	1.44 ± 0.11 h,i,l	1.04 ± 0.01 u,t
Nagazaki (*M. alba* L.)	70.2	3.75 ± 0.11 e	2.94 ± 0.02 f

^a^ Means followed by distinct letters in the same column statistically differ according to Tukey’s post hoc test, (*p* < 0.05). Homogeneous subsets are indicated by the same letter. Two-way factorial ANOVA: Cultivar F_30, 186_ = 1392, *p* < 0.05. Treatment F_1, 186_ = 597, *p* < 0.05. Cultivar*Treatment F_30, 186_ = 34, *p* < 0.05.

**Table 2:** 1-DNJ content of 14 *M. alba* cultivars harvested in 2018. Data are expressed as mg of 1-DNJ/g of dry leaf ± SD.

	End-May 2018		End-July 2018	
*M. alba* Cultivars	Humidity (%)	Dry Samples (mg/g of Dry Matter) ^a^	Humidity (%)	Dry Samples (mg/g of Dry Matter) ^a^
Aobanezumi	67.0	0.38 ± 0.02 l	65.3	0.77 ± 0.02 h,i
Cattaneo female	65.2	0.84 ± 0.02 b	64.0	3.88 ± 0.05 c
Cattaneo male	69.4	0.74 ± 0.03 c	69.0	2.60 ± 0.03 f
Egyptienne	62.2	0.42 ± 0.02 l	59.2	0.74 ± 0.02 h,i
Florio	52.0	0.36 ± 0.01 l	50.3	0.88 ± 0.02 h
Giazzola	68.2	0.53 ± 0.02 i	-	-
Kayriounezumigaeshi	66.1	0.97 ± 0.03 a	65.3	3.48 ± 0.05 d
Limoncina	59.2	0.42 ± 0.02 l	58.8	0.44 ± 0.02 l
Morettiana	74.1	0.74 ± 0.04 c	73.3	2.75 ± 0.09 e
Nervosa	66.0	0.92 ± 0.03 a	65.2	5.01 ± 0.08 b
Pendula	70.2	0.99 ± 0.05 a	68.9	5.48 ± 0.10 a
Pyramidalis	69.3	0.63 ± 0.05 d	68.4	2.20 ± 0.05 g
Spain Black Fruit	74.4	0.97 ± 0.03 a	74.0	2.13 ± 0.05 g
Ukraina	63.8	0.53 ± 0.02 i	62.1	0.65 ± 0.02 i

**^a^** Means followed by distinct letters in the same column statistically differ according to Tukey’s post hoc test (*p* < 0.05), homogeneous subsets are indicated by the same letter. The sample Giazzola relative to the end-July 2018 harvest is missing due to the inability to collect leaves (poor harvest due to bad weather conditions). Two-way factorial ANOVA: Cultivar F_13, 123_ = 2294, *p* < 0.05. Season F_2, 123_ = 16512, *p* < 0.05. Cultivar*Season F_25, 123_ = 1663, *p* < 0.05.

**Table 3:** 1-DNJ content of *Morus alba* cultivars in 2017 and 2018 samplings.

	End-May 2017		End-May 2018	
*M. alba* Cultivars	Humidity (%)	Dry Samples (mg/g of Dry Matter) ^a^	Humidity (%)	Dry Samples (mg/g of Dry Matter) ^a^
Aobanezumi	69.4	0.44 ± 0.06 i	67.0	0.38 ± 0.02 f
Cattaneo Female	66.3	0.59 ± 0.03 h	65.2	0.84 ± 0.02 b
Cattaneo Male	70.5	1.50 ± 0.03 e	69.4	0.74 ± 0.03 c
Egyptienne	52.9	1.66 ± 0.08 d	62.2	0.42 ± 0.02 f
Florio	70.7	0.55 ± 0.02 h,i	52.0	0.36 ± 0.01 f
Giazzola	67.7	1.20 ± 0.05 f	68.2	0.53 ± 0.02 e
Kayriounezumigaeshi	68.1	1.86 ± 0.08 c	66.1	0.97 ± 0.03 a
Limoncina	64.2	0.93 ± 0.05 g	59.2	0.42 ± 0.02 f
Morettiana	72.5	1.26 ± 0.04 f	74.1	0.74 ± 0.04 c
Nervosa	74.4	1.62 ± 0.06 d,e	66.0	0.92 ± 0.03 a
Pendula	72.6	1.43 ± 0.04 e	70.2	0.99 ± 0.05 a
Pyramidalis	70.2	2.07 ± 0.08 b	69.3	0.63 ± 0.05 d
Spain Black Fruit	70.3	1.76 ± 0.07 c,d	74.4	0.97 ± 0.03 a
Ukraina	70.5	2.94 ± 0.06 a	63.8	0.53 ± 0.02 e

**^a^** Means followed by distinct letters in the same column statistically differ according to Tukey’s post hoc test (*p* < 0.05), homogeneous subsets are indicated by the same letter. Two-way factorial ANOVA: Cultivar F_13, 84_ = 593, *p* < 0.05. Year F_1, 84_ = 7615, *p* < 0.05. Cultivar*Year F_13, 84_ = 428, *p* < 0.05.

**Table 4:** Different climatic conditions recorded in May 2017 and 2018 in Padova province (45°24′57″96 N lat., 11°52′58″08 E long.), Italy [34].

Year	Average Radiation (kWh/m^2^/day)	Monthly Total Rainfall (mm)	Average Temperature (°C)
2017	5.70 (20.52 MJ/m^2^/day)	79 mm	18.6
2018	5.16 (18.57 MJ/m^2^/day)	81.8 mm	19.4

**Table 5:** Recovery percentage and precision data obtained with spiked samples.

μg/mL	Intra—Day *n* = 3						
	1	2	3	Mean	SD	RSD (%)	Recovery (%)
0.06	0.056	0.058	0.064	0.059	0.004	7.02	98.89
0.60	0.630	0.550	0.570	0.583	0.042	7.14	97.22
6.00	5.980	6.720	6.400	6.367	0.371	5.83	106.11
	**Inter—Day *n* = 3**						
	**1**	**2**	**3**	**mean**	**SD**	**RSD (%)**	**Recovery (%)**
0.06	0.062	0.058	0.054	0.058	0.004	6.90	96.67
0.60	0.570	0.620	0.580	0.590	0.026	4.48	98.33
6.00	6.120	5.940	6.200	6.087	0.133	2.19	101.44
